# Deriving task specific performance from the information processing capacity of a reservoir computer

**DOI:** 10.1515/nanoph-2022-0415

**Published:** 2022-10-03

**Authors:** Tobias Hülser, Felix Köster, Kathy Lüdge, Lina Jaurigue

**Affiliations:** Institut für Theoretische Physik, Technische Universität Berlin, Berlin, Germany; Technische Universität Ilmenau, Institute of Physics, Ilmenau, Germany

**Keywords:** information processing capacity, memory capacity, nonlinear oscillator, reservoir computing

## Abstract

In the reservoir computing literature, the information processing capacity is frequently used to characterize the computing capabilities of a reservoir. However, it remains unclear how the information processing capacity connects to the performance on specific tasks. We demonstrate on a set of standard benchmark tasks that the total information processing capacity correlates poorly with task specific performance. Further, we derive an expression for the normalized mean square error of a task as a weighted function of the individual information processing capacities. Mathematically, the derivation requires the task to have the same input distribution as used to calculate the information processing capacities. We test our method on a range of tasks that violate this requirement and find good qualitative agreement between the predicted and the actual errors as long as the task input sequences do not have long autocorrelation times. Our method offers deeper insight into the principles governing reservoir computing performance. It also increases the utility of the evaluation of information processing capacities, which are typically defined on i.i.d. input, even if specific tasks deliver inputs stemming from different distributions. Moreover, it offers the possibility of reducing the experimental cost of optimizing physical reservoirs, such as those implemented in photonic systems.

## 1 Introduction

Reservoir computing is a versatile, fast-trainable machine learning scheme inspired by the human brain [1, 2]. It avoids difficulties in the training of recurrent neural networks, like the vanishing gradient in time [3] by using a high-dimensional dynamical system, instead of a network with optimized weights, and only training the linear output weights. Recently, it was shown that the universal approximation property holds for a wide range of reservoir computers [4], demonstrating the generality of the reservoir computing approach. Furthermore, because there is no need to train weights within a reservoir, a wide range of hardware implementations have been shown to be feasible [5–9]. Of particular interest are optical implementations, due to the potential speed up in computation times [10, 11].

Generally, two different approaches to reservoir computing exist. First, the so-called echo state approach, which typically uses a reservoir constructed out of randomly connected nonlinear nodes [1] and has been implemented both experimentally [6, 7, 9, 12] and computationally [13–15]. Second, the alternative delay-based approach introduced in [16], where a single dynamical node, e.g., a laser subjected to external time delayed feedback, serves as a time-multiplexed reservoir. This approach has the benefit of relatively simple implementation and uses the dynamic complexity of time delayed systems [17], introducing so-called virtual nodes. There have been various experimental realizations of the delay-based scheme, including optoelectronic [8, 16, 18, 19], optical [20–22] and electrical [23] ones. Potential applications are time-series-predictions [24, 25], fast word recognition [10], signal conditioning [26] and optical communication [27].

Aside from speed and power consumption issues, one wants reservoir computers (RCs), which perform well on various regression or classification tasks. In order to evaluate the performance of a reservoir computer (RC), there are various benchmark measures, e.g., the very commonly used NARMA10 task. A task-independent measure is the (linear) memory capacity [14], which measures the capability of the reservoir to reconstruct previous inputs. The linear memory capacity was generalized to the information processing capacity by Dambre et al. [28] in 2012, which measures the capability of a reservoir to memorize previous inputs and perform nonlinear calculations on them. The information processing capacities (IPCs) are also referred to as nonlinear memory capacities in the literature. This measure has been used in a number of experimental and theoretical publications [29–32] as a classification of the computing abilities of a reservoir. Besides measuring the IPC in theoretical or experimental frameworks, there are multiple recent advances in calculating and manipulating the IPC. For instance, it was recently possible to calculate the linear part of the IPC (the linear memory capacity) through a linearization of the operating reservoir [33], to systematically manipulate the memorizable inputs via delay-time tuning in a delay-based approach [34] and to manipulate the orders of nonlinear transformation performed via manipulating the input gain [29, 35]. Very recently, the measure of IPC was generalized to systems that are not time invariant [36]. The corresponding measure was introduced as the temporal information processing capacity (TIPC) and has potential relevance for biological systems, as it could be measured in neural cortices [36].

However, despite the extensive research that has been carried out on the IPC, the general connection between the IPC and task-specific performance remains unclear. As an additional challenge, the IPC is typically defined on i.i.d. input; however, tasks often require a different input distribution. Moreover, to the authors’ knowledge, there is no known measure that characterizes a reservoir and at the same time strongly correlates to the performance on specific tasks. It is the aim of this paper to work towards addressing these issues by presenting a method to explicitly relate the IPCs to task-specific performances by providing estimates of a RCs performance on specific tasks using its IPCs. The relevance of this work for hardware-implemented reservoirs, such as photonic reservoirs, is that if the IPC is experimentally determined, our approach would allow for an efficient optimization of the performance of the reservoir on a range of tasks.

This work is structured as follows. First, we shortly explain the concept of reservoir computing, using the example of delay-based reservoir computing, and introduce the information processing capacity, as well as the typical benchmarking tasks used in this work. Second, we motivate our new approach by showing that the commonly used sums of IPCs are in general only weakly correlated to task-specific performance. Third, we analytically derive an explicit relation between weighted sums of IPCs and task-specific performance for the case that the task has the same input distribution as used to calculate the IPCs, thereby obtaining an estimate of a tasks error out of its IPCs. We then analyse the validity of our method when the input deviates from the mathematical constraints.

## 2 Methods

In reservoir computing, a dynamical system, called a reservoir, is fed with input information and the nonlinear response of the reservoir is used to perform a linear approximation of an input-dependent specific task. In the original approach, the reservoir consists of many randomly coupled nonlinear nodes with, e.g., tanh-function dynamics [1]. The input enters into the system via a weighted input matrix. The nonlinear response to the input is then read out via a linear combination of the internal-nodes states. The output weights are trained to minimize the Euclidean distance between the generated output and the target. We refer the reader to the literature [1, 37], [38], [39], [40] for more in-depth discussions of the concepts and mathematical foundations. In the alternative delay-based approach [16], instead of multiplexing in space, the system is multiplexed in time via measuring the systems’ response at multiple times. In the simplest case, one has a system with one dynamical variable *x*(*t*) (“real node”) subjected to a linear delayed feedback term [16]. Several expansions to the original concept with more than one real node were discussed in the literature [41–44].

In this manuscript, we use the delay-based approach, which is shortly introduced in the following. For a more thorough discussion, consider, e.g., [16, 39]. However, our analytical derivations (Section 3) make no reference to what system is used as a reservoir. All of the results are expected to hold in the Echo State Network approach as well.

### 2.1 Time-delayed reservoir computing

**Figure 1:**
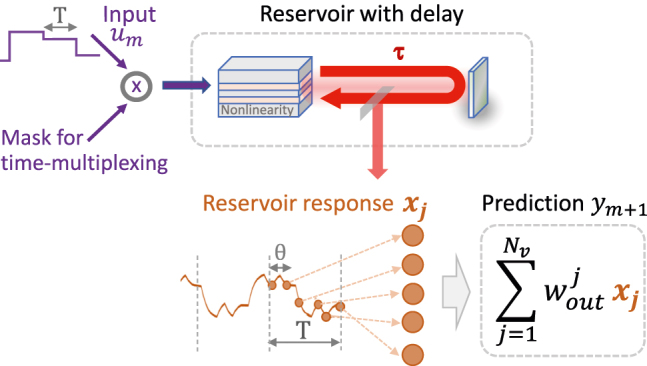
Scheme of a time multiplexed reservoir computer with a nonlinearity (e.g., a laser) subjected to delayed feedback.

In the delay-based approach of reservoir computing [16, 27, 34, 39, 40, 45], a nonlinear system subjected to one or multiple delays is fed by an input series (*u*_1_, *u*_2_, …, *u*_*M*_) and the response of the system is measured multiple times during each input interval. The latter procedure is called time-multiplexing. A corresponding setup is shown in Figure 1. Using a so-called sample-and-hold procedure, each input is fed into the system for an interval *T*, called the input clock cycle. Inside each input interval, a *T*-periodic mask function *g* is applied on the inputs. The mask is typically a stepwise constant function with random step heights. Applying it reduces the linear dependency of the virtual nodes. If the system contains multiple real nonlinear nodes, e.g., a laser network with time-delayed coupling, each node obtains its own input with its own mask function.

The virtual nodes are the states *x*(*t*) evaluated at different times *t* = (*m* − 1)*T* + *jθ*; *m* ϵ [1, 2, …, *M*], *j* ϵ [1, 2, …, *N*_*v*_]. The separation between the virtual nodes is denoted as $\theta =\frac{T}{N_{v}}$ , where *N*_*v*_ is the number of virtual nodes. The output *y*_*m*_ is constructed as a linear combination of the virtual nodes:

(1) $$y_{m}=\sum_{j=1}^{N_{v}}w_{\text{out}}^{j}x\left(\left(m-1\right)T+\theta j\right)+b,$$

with a constant bias *b*. In the training phase, the reservoir is fed *M*_tr_ successive inputs and the response of the reservoir is sampled *N*_*v*_ times for each input. These responses are written into a $M_{\text{tr}}\times \left(N_{v}+1\right)$ -dimensional state matrix **S**, the last column of which is filled with a bias term of one. The linear weights **w**_out_ are trained to minimize a suitable loss function. We choose the Euclidean difference between output **y** = **Sw** ^out^ and target output $\hat{y}$ and train via ridge regression. The ridge regression is the solution to

(2) $$\underset{w_{\text{out}}}{\min}\left(\|Sw_{\text{out}}-\hat{y}{\|}_{2}^{2}+\zeta \|w_{\text{out}}{\|}_{2}^{2}\right).$$

Here, *ζ* denotes the Tikhonov regularization parameter, introduced to avoid overfitting. Using the Moore–Penrose pseudoinverse, the solution to Eq. (2) is given by

(3) $$w_{\text{out}}={\left(S^{T}S+\zeta I\right)}^{-1}S^{T}\hat{y}.$$

To quantify the quality of the prediction, we use the normalized mean-square error (NMSE). It is defined as

(4) $$\text{NMSE}=\frac{\overset{M}{\underset{m=1}{\sum }}{\left(y_{m}-{\hat{y}}_{m}\right)}^{2}}{M\cdot \text{var}\left(\hat{y}\right)}.$$

here $y_{m}\left({\hat{y}}_{m}\right)$ denotes the *m*th prediction (target) value, *M* denotes the number of samples and $1/\text{var}\left(\hat{y}\right)$ is a normalization factor. Zero NMSE indicates a perfect prediction, and one corresponds to $y_{m}=\text{mean}\left(\hat{y}\right)$ .

When the reservoir contains multiple real nodes *x*_1_(*t*), *x*_2_(*t*), …, each real node contains its own virtual nodes and the state matrix **S** is expanded accordingly. The reservoir used in this manuscript is described in Section 4.

### 2.2 Information processing capacity

The information processing capacity is a task-independent method of characterizing the performance of a reservoir computer. It quantifies the capability of the reservoir to reconstruct a set of basis functions in the Hilbert space of fading memory functions [28] and is a generalization of the concept of linear memory capacity [14]. In the following, we give a brief introduction to the IPC, using definitions suitable for the later calculations. As mentioned before, the corresponding measures are also referred to as nonlinear memory capacities. Generally, the capacity to reconstruct a certain task $\hat{y}$ is defined as [28]

(5) $$C_{\hat{y}}≔1-\frac{\text{MSE}\left(y\right)}{1/M\|\hat{y}{\|}^{2}},$$

where $y={\left(y_{1},y_{2},\ldots y_{M}\right)}^{T}=Sw$ is the best linear approximation of a target vector $\hat{y}$ and MSE denotes the mean square error. Note, that the capacity is evaluated without a regularization, i.e., *ζ* = 0. In [46], it is shown that the capacity is more conveniently given by

(6) $$C_{\hat{y}}=\frac{{\hat{y}}^{T}y}{\|\hat{y}{\|}^{2}}.$$

If the capacity equals 1, the approximation is perfect, i.e., $y=\hat{y}$ . If the capacity is zero, the system is not at all capable of linearly estimating **y**. In between, a partial reconstruction is possible. The IPCs are the capacities, described by Eq. (6), to reconstruct individual basis functions **P**_*n*_ on the Hilbert space of fading memory functions, i.e., $\hat{y}=P_{n}$ .

In order to construct a basis on the Hilbert space of fading memory functions, first consider the sequence {*u* ^−*h*^} = {*u*_0_, *u*_−1_, …, *u*_−*h*_} of a current input *u*_0_ and *h* previous inputs prior to *u*_0_. Then, one can construct a basis out of finite products of Legendre polynomials *p*_*d*_(*u*_−*i*_) [28], where *u*_−*i*_ denotes the input *i* time steps into the past and *p*_*d*_(*u*_−*i*_) denotes the Legendre polynomial of order *d* corresponding to input *i* time steps into the past. The resulting basis functions are *P*_*n*_(*u* ^−∞^), with $P_{n}\left(u^{-\infty }\right)=\Pi_{i}p_{d_{n}^{i}}\left(u_{-i}\right)$ . Here, the index *n* = {*d* ^*i*^} = {*d* ^1^, *d* ^2^, …, } serves as a multi-index corresponding to a set of degrees *d* ^*i*^, only finite of which are non-zero. Formally, the memory capacity is defined for an infinitely long input sequence, i.e., *h* → ∞, but for numerical evaluation, a suitable cut-off has to be chosen (see Supplementary material for details). As input sequence, we use independent and identically distributed (i.i.d.) random numbers in [−1, 1]. In [28], it is shown that the so defined collection of *P*_*n*_ indeed form an orthogonal basis on the Hilbert space of fading memory functions. The IPCs to reconstruct a collection of *P*_*n*_ are now given via Eq. (6):

(7) $$\begin{array}{ll} IPC_{n} & ≔C_{\hat{y}=P_{n}}=\frac{P_{n}^{T}y}{\|P_{n}{\|}^{2}}\\ P_{n}\left(u^{-\infty }\right) & =\Pi_{i}p_{d_{n}^{i}}\left(u_{-i}\right) \end{array}$$

We can define the order *d*(*P*_*n*_) of a basis element *P*_*n*_ as the sum of the orders of the individual capacities in the sequence {*d* ^*i*^} yielding *d*(*P*_*n*_) = *∑*_*i*_ *d* ^*i*^. The degree is used to define linear, quadratic, and higher order capacities as the sum of all capacities corresponding to a certain degree. Using Eq. (7), the IPC of degree *d* is defined as

(8) $$IPC^{d}=\underset{n:\;d\left(P_{n}\right)=d}{\sum }IPC_{n},$$

where the summation takes place over all capacities corresponding to basis elements *P*_*n*_ with order *d*(*P*_*n*_) = *d*.

Finally, the total IPC is defined as the sum

$${\text{IPC}}^{T}=\underset{d>0}{\sum }IPC^{d}.$$

In [28], it was shown that the total information processing capacity is always smaller than or equal to the output dimension, i.e., the number of virtual nodes.

### 2.3 Benchmark tasks

Four standard benchmark tasks are considered in the study: NARMA10 [47], one-step-ahead predictions of the *x* variable of the Lorenz 63 system [48], nonlinear channel equalization and one-step-ahead prediction of the Mackey–Glass system [49]. Descriptions of all four tasks can be found in Section 1 of the Supplementary material.

### 2.4 Reservoir – network of ring coupled Stuart–Landau oscillators

**Figure 2:**
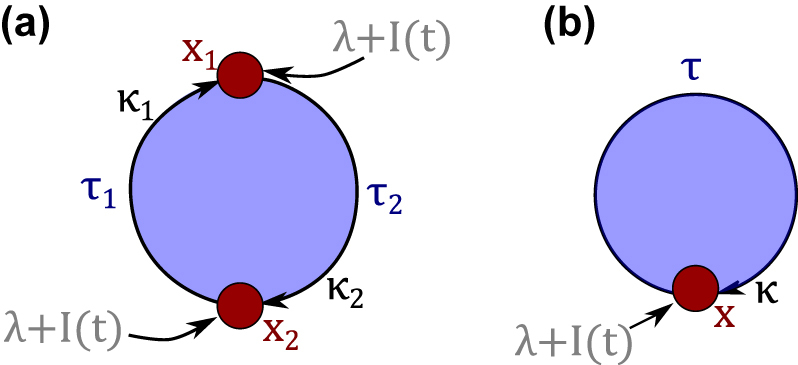
Delay-coupling schemes used for the reservoir: (a) Two oscillators *x*_*j*_ arranged in a delay-coupled ring topology, corresponding to Eq. (9) where each oscillator is unidirectionally coupled to the next oscillator with delays *τ*_*j*_. Every node is externally driven with pump rate *λ*_*j*_. The input *I*(*t*) = *g*_*j*_(*t*)*u*(*t*) is fed in via the pumping. (b) Same setup as in (a), but with one oscillator subjected to delayed feedback.

As a numerical testing setup, we use a system of delay coupled Stuart–Landau oscillators in a delayed ring topology modeled by

(9) $$\begin{array}{ll} {\dot{x}}_{j} & =\left(\lambda_{j}+\eta_{j}g_{j}\left(t\right)u\left(t\right)+i\omega_{j}+\left(\gamma_{j}+i\alpha_{j}\right)|x_{j}{|}^{2}\right)x_{j}\\ & \;+\kappa_{j}{\text{e}}^{\text{i}\phi_{j}}x_{j+1}\left(t-\tau_{j}\right)+D_{\text{noise}}\xi \left(t\right), \end{array}$$

where *λ*_*j*_ denotes the pump rate of oscillator *j*, *ω*_*j*_ the frequency, *γ*_*j*_ the nonlinearity parameter, *α*_*j*_ denotes the re-scaled sheer parameter, *κ*_*j*_ the feedback strength, *ϕ*_*j*_ the feedback phase, *τ*_*j*_ the delay time and *ξ*(*t*) models Gaussian white noise with amplitude *D*_noise_ = 10^−8^. If the number of oscillators is *N*, then the ring topology implies *x*_*N*+1_ ≡ *x*_1_. The input is denoted as *η*_*j*_ *g*_*j*_(*t*)*u*(*t*), where *u*(*t*) is the original (unmasked) input, *g*_*j*_(*t*) denotes the *jth* mask function and *η*_*j*_ the input strength. The mask functions were chosen to be piecewise constant random binary. It is important to note that each oscillator *j* has a distinct mask. Figure 2a illustrates the oscillator setup with two nodes (*j* ϵ {1, 2}) and Figure 2b with one node (*j* = 1). The system is similar to a system of coupled lasers, as the Stuart–Landau oscillator is the generic form of a Hopf bifurcation and behaves like a laser near the lasing threshold [31]. The parameters chosen for the simulations are as in Table 1, unless stated otherwise.

**Table 1:** Default parameter values for numerical simulations.

Parameter	Description	Value
*τ*	Feedback delay time	425
*T*	Clock cycle	200
*Λ*	Pump rate	0.01
*κ*	Feedback strength	0.18
*Φ*	Feedback phase	0
*η*	Input strength	0.06
*α*	Sheer parameter	0
*ω*	Frequency	0
*γ*	Nonlinearity	−0.1
*N*_*v*_	Virtual nodes per oscillator	100

## 3 Connection between information processing capacity and task-specific performance

In the literature, in the context of the IPC, mostly measures consisting of sums of capacities of a certain order are investigated (*IPC* ^*d*^ in Eq. (8)). However, the summed *IPC* ^*d*^s are in general only weakly correlated to the error of a specific task and are, therefore, a poor measure for predicting task-specific performance. As an example, we consider a system of two delay-coupled Stuart–Landau oscillators in a ring coupling configuration (see Section 2.4 for details). In this setup, we find that the ring delays have a strong influence on the IPCs and task performance, but the parameter dependencies of these quantities vary strongly.

**Figure 3:**
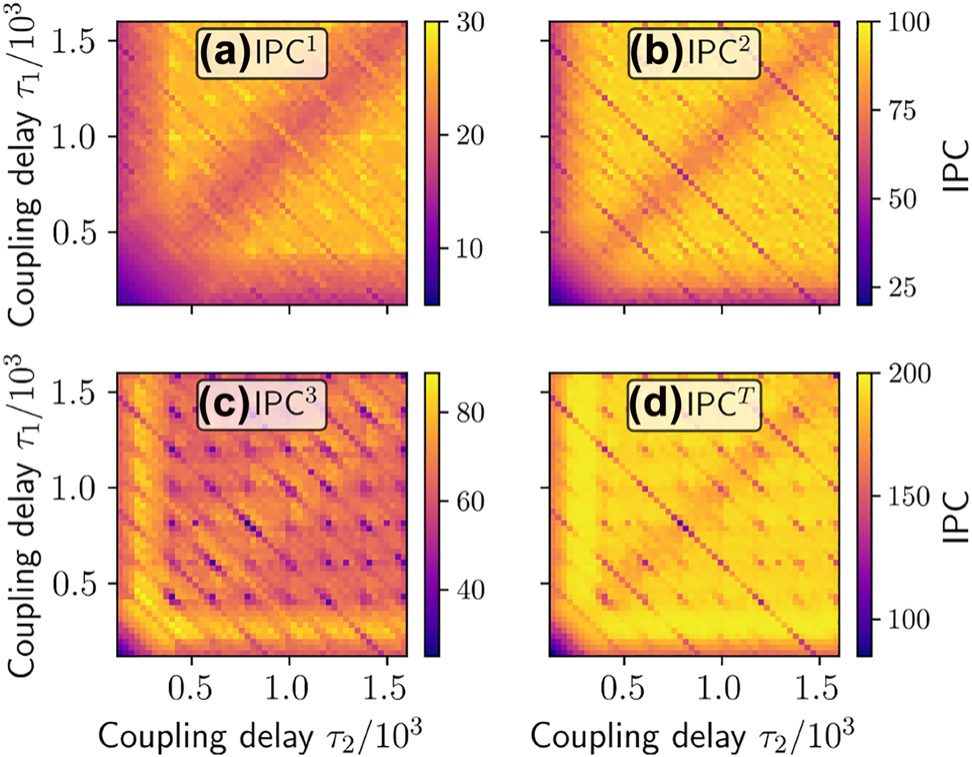
Information processing capacities: (a) IPC^1^, (b) IPC^2^, (c) IPC^3^ and (d) IPC^*T*^, as defined in Eq. (8), plotted colour coded as a function of *τ*_1_ and *τ*_2_ in the ring-coupled system Eq. (9) with two oscillators. Pearson correlation coefficients between the IPCs and the task NMSEs shown in Figure 4 are given in Table 2.

**Figure 4:**
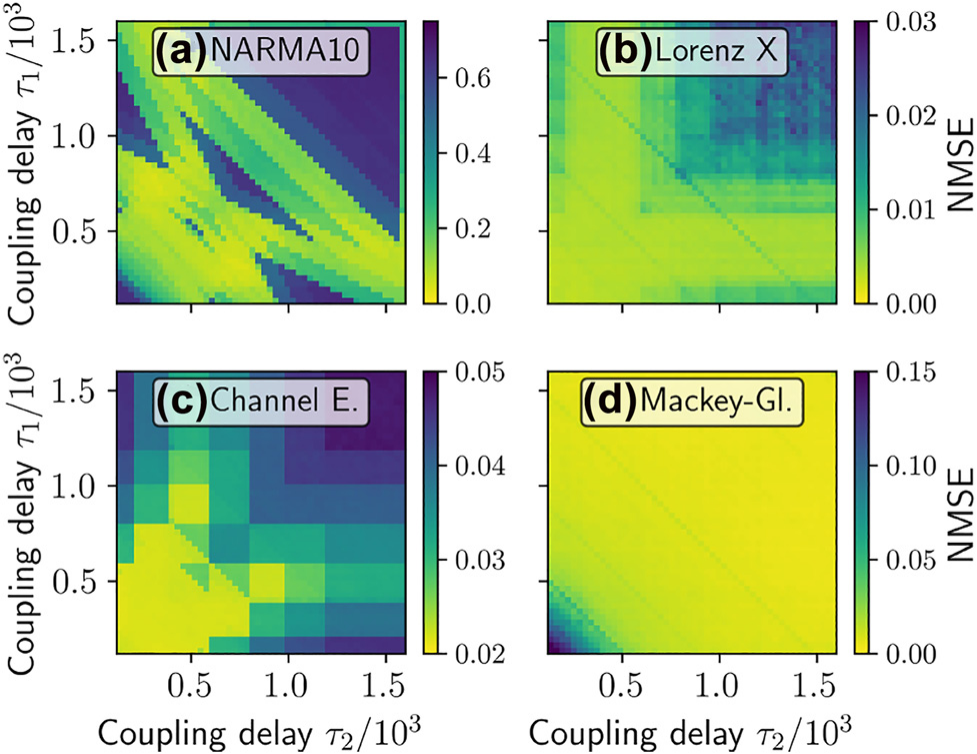
Performance on different tasks: (a) NARMA10, (b) Lorenz x, (c) channel equalization and (d) Mackey–Glass task errors (NMSE) plotted colour coded as a function of *τ*_1_ and *τ*_2_ in the ring-coupled system Eq. (9) with two oscillators. Pearson correlation coefficients between the task NMSEs and the IPCs shown in Figure 3 are given in Table 2.

In Figure 3, a scan over the two ring delays *τ*_1_, *τ*_2_ is performed and the linear, quadratic, cubic and total IPC are depicted. Figure 4 shows the corresponding plots for the performance on various regression benchmark tasks. For all tasks, a strong dependence on the delay times is evident and various resonance lines can be found, as explained in [34]. However, important for the predictive power of the summed *IPC* ^*d*^s is that the delay dependence is different for each of the tasks.

To quantify the predictive power of the summed *IPC* ^*d*^s, we calculate a Pearson correlation coefficient between the depicted *IPC* ^*d*^ *s* and the NMSE of the benchmark tasks. A negative value close to −1 indicates a low NMSE at values of high IPC, whereas a high positive value indicates a counterproductive effect between the memory capacity and the task NMSE. Zero indicates no linear statistical correlation between both quantities. As one can see in Table 2, the obtained correlation coefficients are generally low. Furthermore, some values are positive, meaning that high values of the corresponding sum of IPCs tend to correspond to low task performance. These results demonstrate that choosing reservoir hyperparameters based on summed capacities, such as the total IPC, is not helpful for optimizing task-specific performance. The underlying reason is that most tasks have specific IPC requirements and do not need all available ones. Different past inputs are in general not equally relevant for the performance of a specific tasks. A simple sum of all IPCs is, therefore, a very limited, yet often considered, measure.

**Table 2:** Pearson correlation coefficients between the information processing capacities (IPC) (plotted in Figure 3) and the benchmark task NMSE errors (plotted in Figure 4).

Task	*IPC* ^1^	*IPC* ^2^	*IPC* ^3^	Total IPC
Lorenz X	0.18	0.12	−0.20	−0.07
NARMA10	0.07	0.03	−0.13	−0.08
Channel equal.	0.14	0.08	−0.12	−0.02
Mackey–Glass	−0.62	−0.63	−0.02	−0.53

### 3.1 Analytic derivation of an explicit relationship between IPCs and NMSE

The appeal of the information processing capacity is that it quantifies the computational capabilities of a reservoir in a task-independent manner. In this section, we introduce a method of connecting the IPCs with task-specific performance. The motivation for this is that once the IPCs of a reservoir are determined, these can be used to predict the performance of the reservoir on a multitude of tasks. This can reduce the hyperparameter optimization, which would otherwise need to be carried out individually for each task.

To generate a more task relevant measure than simple sums of information processing capacities, we build a weighted sum of IPCs instead of the unweighted sums usually considered. The corresponding ansatz is

(10) $$IPC_{\text{task}}=1-\underset{n}{\sum }c_{n}\cdot IPC_{n},$$

where $c_{n}\in R$ denotes the weight of the *n*th capacity and n∈N serves as a (multi-)index, indexing all possible capacities, as in Section 2.2. The above approach is justified analytically in the following. The values of *c*_*n*_, that are derived, give the information, which capacities are relevant for a given task. Note that until now, there only exist some approaches to directly evaluate relevant IPCs of the NARMA10 task [36] or indirectly evaluate the previous inputs that are relevant to specific tasks [50]. However, a direct connection between IPC and RC performance was not described yet.

The exact calculations are shown in the Supplementary material, here only the most important steps are given. Consider the capacity to compute an arbitrary task (Eq. (6)) [46]:

(11) $$\begin{array}{ll} C_{{\hat{y}}_{n}} & =\frac{{\hat{y}}_{n}^{T}y}{\|\hat{y}{\|}^{2}} \end{array}$$

The idea is to develop the task $\hat{y}$ into a basis {*P*_*n*_} on the Hilbert space of fading memory functions and simultaneously develop the prediction into a series of predictions of the basis elements *P*_*n*_ with identical development coefficients. Note, if one instead directly develops the prediction into a basis on the Hilbert space of fading memory functions, one obtains a direct relationship between reservoir computing and nonlinear vector autoregressive models (see Supplementary material).

It is known that any time invariant dynamical system with fading memory can be approximated by a finite discrete Volterra series [51]. The discrete Volterra series is a series of products of monomials of previous inputs. We, therefore, assume that the task $\hat{y}$ is time invariant and has fading memory property. For the prediction, this is ensured by the fading memory property of reservoir computing. The Volterra series is non-orthogonal and, therefore, not suitable for the purpose of this paper. However, it is possible to choose an orthogonal basis dependent on the input distribution, as it is done with products of Legendre polynomial in the definition of the IPC Eq. (6). This connects to a technique called polynomial chaos expansion, where a random variable is expanded in a polynomial basis orthogonal on a possibly arbitrary input distribution [52]. Polynomial chaos expansions have, e.g., been successfully used in a context of uncertainty quantification [52, 53], they are not limited to fading memory systems and converge under general conditions [52, 54].

We can, therefore, expand the target function $\hat{y}$ as

(12) $$\hat{y}=\underset{n}{\sum }a_{n}P_{n}+\xi ,$$

where *P*_*n*_ are the basis functions, *a*_*n*_ the corresponding coefficients, and *ξ* is a noise term independent of the input. We set *P*_0_ = 1 and, therefore, *a*_0_ is the constant bias term. The choice of (useful) basis functions is dependent on the probability distribution of the input series. In the context of IPC, often identically drawn independent random numbers in [−1, 1] are chosen for the input. In this case, an orthogonal set of basis functions are products of Legendre polynomials formed by different past input steps (see Section 2.2). We use this basis in Section 4, but the analytical calculations are generalizable and do not refer to a specific basis. We emphasize that other orthogonal bases could be used as well, see e.g., [36], for use of different bases in an IPC context.

While we developed the target into the above-mentioned series (Eq. (12)) and obtain the $a_{n}^{\prime }s$ , we can develop the prediction into predictions of the corresponding basis functions via

(13) $$y=\underset{n}{\sum }a_{n}p_{n}+h\left(\xi \right),$$

where *p*_*n*_ is the prediction of basis function $P_{n}$ within a given input series and *h*(*ξ*) is a noise term, stemming from the input-independent part of the target series. It will be neglected in the following. Note that the development coefficients *a*_*n*_ are the same as in the series expansion Eq. (12) (see Supplementary material).

We further assume that the input series for the task comes from the same probability distribution as in the definition of the IPC, for example i.d.d. random numbers in [−1, 1]. If one then puts Eqs. (12) and (13) into Eq. (11), we obtain a formula for the NMSE for a given task:

(14) $$NMSE_{\text{pred}}\approx 1-\frac{\underset{n}{\sum }a_{n}^{2}\|P_{n}{\|}^{2}IPC_{n}}{N\text{var}\left(\hat{y}\right)}.$$

Here, *a*_*n*_ are the coefficients of the series expansion of the task (Eq. (12)), ‖**P**_*n*_‖^2^ the squared norms of the basis polynomials and $N\text{var}\left(\hat{y}\right)$ the variance of the target $\hat{y}$ multiplied with the number of training samples *N*. *IPC*_*n*_ denotes the *n*th information processing capacity corresponding to basis polynomial *P*_*n*_. For simplicity, it is assumed that the mean of the target equals zero; however, non-zero mean values are taken care of via the bias term. Eq. (14) is, therefore, to be evaluated without the constant bias term, i.e., *a*_0_ = 0.

In order for Eq. (14) to be used, the expansion coefficients *a*_*n*_ must be determined. To evaluate the coefficients *a*_*n*_, we use a linear regression approach, constructing a linear model out of the basis elements *P*_*n*_(*u* ^−∞^). The corresponding model is

(15) $$y=\underset{n}{\sum }{\tilde{a}}_{n}P_{n}\left(u^{-\infty }\right).$$

Where **y** denotes the prediction of the target vector $\hat{y}$ . The coefficients ${\tilde{a}}_{n}$ are evaluated via ridge regression and serve as an estimate for the searched coefficients *a*_*n*_. Details can be found in the Supplementary material. This is a nonlinear vector regressive model and similar to a recent approach to predict the Lorenz task [55]. Note, that if the task is an auto-prediction task, e.g., in the Lorenz X task example, the model is a nonlinear vector autoregressive model (NVAR). This approach works well if the regressors *P*_*n*_(*u* ^−∞^) are weakly correlated, i.e., the auto-correlation of the inputs is low, otherwise the model suffers from multicollinearity and the *a*_*n*_ no longer uniquely identifiable. Details on the model can be found in the Supplementary material. It is to be noted that generally, the number of coefficients to be evaluated grows exponentially with increasing order of basis functions and steps into the past considered. Suitable cut-offs, therefore, have to be chosen to limit computational cost and avoid overfitting issues (details are given in Section 4 and in the Supplementary material).

Our approach predicts the NMSE corresponding to a task $\hat{y}$ out of the *IPC*s of the RC. It is important to note that the prediction does not need any knowledge about the RC system other than its IPCs. It is, therefore, not limited to a specific reservoir computing scheme. The main assumption of our derivation of Eq. (14) is that the input for a specific task comes from the same probability distribution as in the evaluation of the IPCs. This assumption does not hold for most real tasks. Additionally, the method used to evaluate the coefficients *a*_*n*_ is best suited for inputs, which are only weakly correlated. Therefore, in Section 4, we test the extent to which Eq. (14) can provide accurate error estimates when these assumptions are not fulfilled.

## 4 Numerical evaluation

In this section, we check the validity of Eq. (14) derived in the previous section.

### 4.1 Impact of deviating from an i.i.d. input distribution

In order to derive the explicit expression relating the NMSE of a task to the IPCs, the assumption was made that the task input distribution equals the IPC input distribution. It is possible to evaluate IPCs for arbitrary input distributions [36] and, therefore, matching arbitrary tasks. However, the IPC input distribution has to be predefined when measuring the IPCs. Therefore, we investigate how well Eq. (14) performs under systematic deviations of this assumption. To do this, we require a task where the input distribution can gradually be altered from i.i.d. random inputs in [−1, 1]. We use the NARMA10 task (given in the Supplementary material). It can be operated with non i.d.d. random inputs, linearly transformed to be in [−1, 1], without having to change the equation that defines the target series.

There are two main factors that are to be considered. First, it is assumed that different inputs *u*_*i*_, *u*_*j*_, *i* ≠ *j* are not correlated, and therefore, the autocorrelation function of the input series {*u*_0_, *u*_1_, …} is zero. This is a severe restriction, since most realistic input series will have an auto-correlation function that is non-zero. Second, it is assumed that the inputs *u*_*i*_ are drawn from a uniform probability distribution *p*(*u*_*i*_). Deviating from a uniform distribution, even in the absence of a non-zero auto-correlation, could also degrade the predictive power of Eq. (14). We analyse both effects with input defined via the following equations:

(16) $$u_{i}=\rho u_{i-1}+\left(\rho -1\right)\chi_{i},$$

where *ρ* denotes a correlation parameter and *χ*_*i*_ denotes independently drawn random numbers from a probability distribution *p*(*χ*). With this construction, *ρ* = 0 corresponds to uncorrelated fully independent *u*_*i*_ with zero auto-correlation and *ρ* = 1 corresponds to *u*_*i*_ = *u*_*i*−1_ and thereby the auto-correlation equals one for every time lag. We can feed the above defined input series *u*_*i*_ into the iterative formula for the NARMA10 task.

To test the predictive power of Eq. (14), we consider the system of one Stuart–Landau oscillator with delayed feedback (see Figure 2b). While fixing the input clock cycle, we scan over the delay time for 50 linearly distributed delays, in a range where the NARMA10 NMSE varies strongly (*τ* ϵ [20, 1600]). This means, we investigate 50 different reservoirs and evaluate the performance of our new method for the NARMA task with different input distributions. We evaluate the Pearson correlation coefficient as well as the mean square error between the predicted NMSE from Eq. (14) and the simulated NMSE.

**Figure 5:**
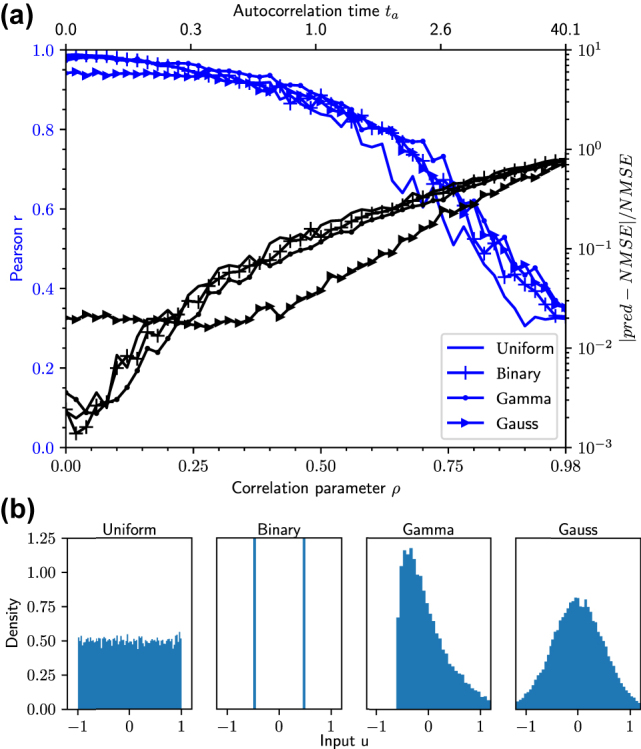
Impact of correlated inputs for performance predictions: (a) Blue lines: Pearson correlation coefficient r between predicted and measured NMSE as a function of the input correlation parameter (see Eq. (16)) for four different random distributions *p*(*χ*) (different line styles). *r* is determined from a scan over 50 distinct delay times *τ* ϵ [20, 1600] in the setup corresponding to Figure 2b. Black lines: Mean square error between predicted and measured NMSE. (b) Histograms of uniform, binary, gamma and Gaussian distribution chosen in (a).

Figure 5a shows these quantities in a scan over the correlation parameter *ρ* for different distributions *p*(*χ*) (different symbols). The line without symbols is the uniform distribution in [−1, 1], where *ρ* = 0 corresponds to i.i.d. inputs and, therefore, the assumptions made in the derivation of Eq. (14) hold (a correlation parameter of *ρ* = 1 corresponds to identical inputs and, therefore, an infinite auto-correlation time). It can be seen that for low *ρ*, Eq. (14) works well. For increasing *ρ*, the mean squared deviation between predicted and measured NMSE becomes increasingly large and the Pearson correlation coefficient decreases. The lines with symbols in Figure 5a show the results for binary, gamma and Gaussian probability distributions *p*(*χ*). We chose a binary distribution between 0 and 0.42, a gamma distribution with shape 1.5 and scale 0.1, and a Gaussian distribution with zero mean and standard deviation 0.3. Note, after the target series is created, the input is linearly normalized such that it approximately fits in the interval [−1, 1], see Supplementary material for further details. Even though these distributions strongly violate the assumption of uniform input, the method works well if *ρ* does not become too high. As a rule of thumb, if one sets a threshold of a Pearson correlation coefficient of at least 0.5, this roughly corresponds to values of *ρ* ⪅ 0.8 (which corresponds to an auto-correlation time *t*_*a*_ ⪅ 4.9, using the definition given below in Eq. (17)).

The results indicate that Eq. (14) is able to qualitatively predict a reservoir computer′s performance on a task, even if the input distribution is not uniform and uncorrelated. For high auto-correlation times, however, Eq. (14) with weights evaluated with the multilinear model given by Eq. (15) performs insufficiently.

### 4.2 Numerical results for benchmark tasks

**Figure 6:**
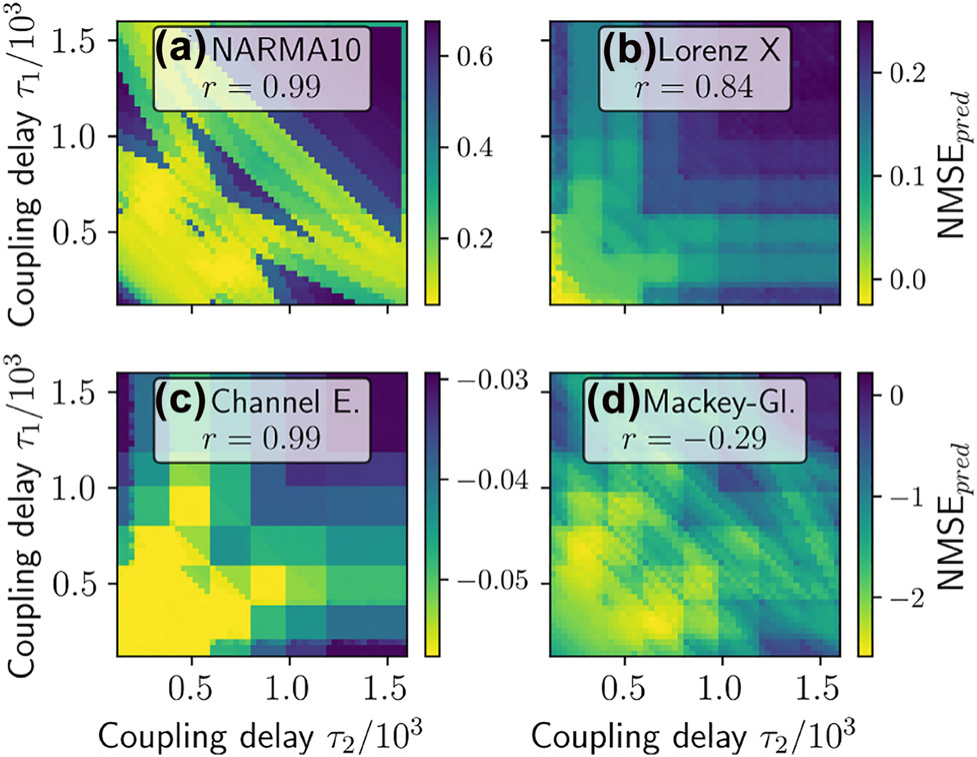
Predicted performance (NMSE) in parameter space for different tasks: (a) NARMA10, (b) Lorenz X, (c) Channel equalization and (d) Mackey–Glass tasks as a scan over the delays in the ring-coupled system of Figure 2a as determined using Eq. (14). (a–c) Insets give Pearson correlation coefficients *r* to the computed errors in Figure 4.

In this section, we test our method on a set of benchmarking tasks. Here, we once again use the system of two delay-coupled Stuart–Landau oscillators in a ring coupling configuration (see Section 2.4 for details) as the test reservoir. In this setup, we obtain complex patterns for the NMSE of the benchmark tasks, if we scan over the two delay times *τ*_1_, *τ*_2_ (Figure 4a–d). In Figure 6a–d, we apply our new method (Eq. (14)) for the NARMA10, Lorenz X, channel equalization and Mackey–Glass tasks. The prediction of the NARMA10 NMSE nearly perfectly reproduces the structure of the simulated NARMA10 NMSE without any knowledge of the system other than the IPCs, which is to be expected in this case as the assumptions made to derive Eq. (14) hold for the NARMA10 input. The Pearson correlation coefficient between the predicted and simulations NMSE is depicted in the respective insets of Figure 6. It reaches a value of 0.99 for NARMA10. In this case, we evaluated capacities up to 2nd order and up to 20 previous inputs. See Supplementary material for a discussion of the cut-off orders.

**Figure 7:**
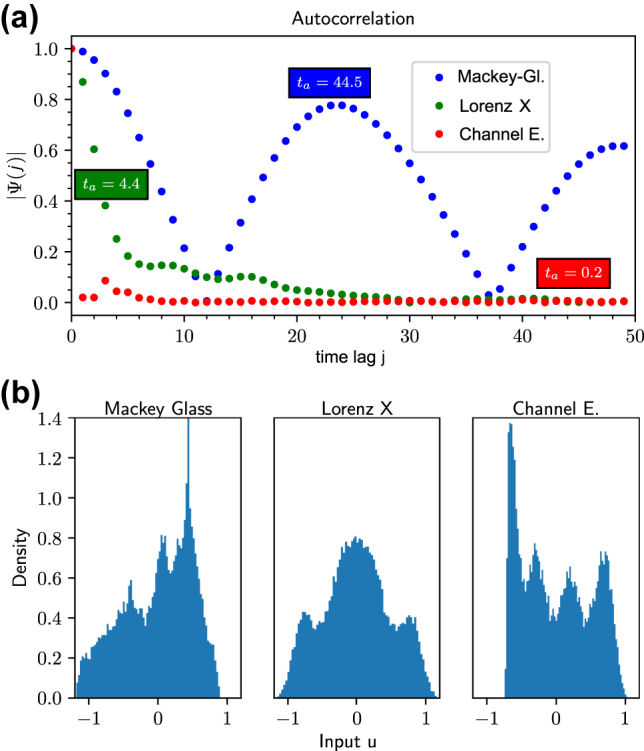
Properties of the input fed into the reservoir: (a) Modulus of the auto-correlation function *Ψ*(*j*) for Lorenz X, Mackey–Glass and channel equalization task, respectively, as a function of the time lag *j* (colored boxes give the auto-correlation times *t*_*a*_ according to Eq. (17)). (b) Histograms of the input distributions for three different tasks.

For the other benchmark tasks evaluated in Figure 6b–d, the assumption of i.i.d. distributed input does not hold. That is first because these tasks have non-zero auto-correlation time (Figure 7a) and second, the distribution of the inputs strongly deviates from an uniform input distribution (Figure 7b). However, if we apply Eq. (14) for the Lorenz task, the structure is still well reproduced (Figure 6b), with a high correlation coefficient of 0.84. For the Lorenz task, we evaluated capacities up to 5th order and up to 5 steps into the past. For the channel equalization task (Figure 6c), the absolute values of the predicted NMSE are inaccurate, in this case even negative. However, the correlation between the predicted and true NMSE is 0.99, a near maximum value. Therefore, for parameter optimization purposes, the predicted NMSE is usable. For the channel equalization task, we evaluated capacities up to third order and up to 10 steps into the past. Both, the Lorenz and the channel equalization task indicate that Eq. (14) is useful even in the case the assumption of i.i.d. input is not fulfilled. These results show that evaluating IPCs for an i.i.d. input distribution still gives information on the performance of a RC fed with inputs from a different distribution.

If we consider the Mackey–Glass task (Figure 6d), the NMSE is poorly approximated using Eq. (14) with coefficients *a*_*n*_ corresponding to Eq. (15). This is because, in comparison with the other tasks, the Mackey–Glass input shows a high input auto-correlation (Figure 7a), which leads to an ambiguous development of the coefficients *a*_*n*_, as well as a larger deviation from the i.i.d. input assumption. For the Mackey–Glass task, we evaluated capacities up to 20 previous inputs and up to 3 orders.

To compare the timescales over which the various inputs are auto-correlated, we calculate the auto-correlation time *t*_*a*_ of the input series according to

(17) $$t_{a}=\overset{j_{\max}\;}{\underset{j=1}{\sum }}|\psi \left(j\right)|,$$

where *ψ*(*j*) is the value of the normalized auto-correlation function at time lag *j* (see Supplementary material for details). To avoid summing up noise, we only evaluated auto-correlation values greater than a threshold of 0.02 and evaluated *j*_max_ = 100 time lags. For the NARMA10 task, we obtain a value of *t*_*a*_ ≈ 0 (i.i.d input), for the Lorenz task *t*_*a*_ ≈ 4.4, for the channel task *t*_*a*_ ≈ 0.2 and for the Mackey–Glass task a value of *t*_*a*_ ≈ 44.5. This indicates a great difference in auto-correlation times *t*_*a*_ between the Mackey–Glass task and the other considered tasks. In Section 4.1, we systematically tuned the auto-correlation of a NAMRA10 input in order to investigate how deviations of i.i.d. input harm the performance of Eq. (14). There, the Pearson correlation between the estimated and the actual NMSE was above 0.5 for *ρ* up to 0.8, which corresponded to an auto-correlation time *t*_*a*_ ⪅ 4.9. This auto-correlation time is similar to the Lorenz task example.

## 5 Conclusions

We have investigated the relationship between the commonly used information processing capacity and the performance on regression tasks, e.g., time series prediction tasks, in reservoir computing. To characterize the computational capabilities of a reservoir, the total information processing capacity or the IPCs summed over each order are commonly considered. However, these simple sums of IPCs are an insufficient measure for predicting task-specific performances. We analytically derived an explicit relation between weighted sums of individual information processing capacities and the expected computing error for specific tasks. We have further tested the extent to which the expression for the NMSE that we have derived deviates from the true NMSE when the problem deviates from the strict mathematical assumptions, i.e., the input distribution of the task varies from that used to characterize the IPC. We found high correlation between the predicted and the actual NMSE for NARMA10, channel equalization and Lorenz time series prediction tasks. For the Mackey–Glass task, we found poor agreement. Our results indicate that the auto-correlation time of the input sequence is crucial in determining the accuracy of the trends of the predicted NMSE, with longer auto-correlation times reducing the applicability of our proposed method.

The above derived approach can be exploited to reduce the experimental cost of optimizing a reservoir computing setup, such as a photonic reservoir, for multiple tasks. Before, in order to obtain measures of the performance for different tasks, the input series for each task had to be fed into the reservoir to measure the responses. With Eq. (14), however, one obtains a direct link between the performance of a task and the individual IPCs of a reservoir computer, and the IPCs can be measured via only one sufficiently large input series and post-processing. By establishing a link between the IPC and the performance for specific tasks, we have demonstrated the utility of the IPC as a means of characterizing physically implemented reservoir computing setups.

## Supplementary Material

Supplementary Material Details
