# Supplementary material for: Deriving task specific performance from the information processing capacity of a reservoir computer
Source: Nanophotonics. 2022 Oct 3;12(5):937–47. doi: 10.1515/nanoph-2022-0415 (PMC11501742; doi:10.1515/nanoph-2022-0415)
Supplement: Supplementary file 1 — Supplementary Material Details [file j_nanoph-2022-0415_suppl.pdf]

Supplementary: Deriving task specific performance from the information processing capacity of a reservoir computer

1. BENCHMARK TASKS

A. NARMA10 task

The nonlinear autoregressive-moving average 10 task (NARMA10) [1] is an often used benchmark in reservoir computing. The task is given by predicting the next time step of the following recursive formula

$$A_{m+1} = 0.3A_m + 0.05A_m \left(\sum_{j=0}^{9} A_{m-j} \right) + 1.5u_{m-9}u_m + 0.1, \tag{S1}$$

where A_m is the iterative NARMA10 time series and u_m are i.i.d. random numbers in [0,0.5] serving as input. Note, that in Sec. 4.1, we alter the distribution of input numbers u_m, in order to test the relation presented in Sec. 3.1.

B. Lorenz X task

The Lorenz task is a time series prediction task. The time series is given by the evolution of the Lorenz attractor, fulfilling the equations [2]

$$\begin{aligned} \dot{X} &= a(Y - X) \\ \dot{Y} &= X(b - Z) - Y \\ \dot{Z} &= XY - cZ. \end{aligned} \tag{S2}$$

There are several variants of the task. We use the X variable, discretised with a time step of $\Delta t = 0.1$, as input and target time-series and predict the next value of this series. As parameters, we use the common values $a = 10, b = 28, c = 8/3$. For initial conditions, we use $X_0 = 0.5, Y_0 = 0.1, Z_0 = 0.2$ and cut off a transient time of $t_{tr} = 100$ before sampling the time series.

C. Mackey Glass task

The Mackey-Glass equation is a delay differential equation exhibiting chaotic dynamics. The benchmarking task is to predict future values of the timeseries. The Mackey-Glass equation is [3]:

$$dx/dt = \beta \frac{x(t-\tau)}{1 + x^n(t-\tau)} - \gamma x \tag{S3}$$

We use the standard parameters: $\beta = 0.2, \tau = 17, n = 10, \gamma = 0.1$. The time series generated by Eq. (S3) is sampled with a time step of $dt = 1$. The target series is to predict the value 10 dt steps into the future. We cut off the first 1000 time-series values as a buffer.

D. Nonlinear Channel Equalisation

We use a variant of the Nonlinear Chanel Equalisation task. This task consists of reconstructing an i.i.d. random sequence of symbols $d(n) \in \{-3, -1, 1, 3\}$ that undergoes a nonlinear propagation channel. The channel is modeled by a linear system of memory length 10 with subsequent nonlinear distortion. The linear channel $q(n)$ is modeled by[4]

$$\begin{aligned} q(n) = {} & 0.08d(n+2) - 0.12d(n+1) + d(n) + 0.18d(n-1) \\ & - 0.1d(n-2) + 0.091d(n-3) - 0.05d(n-4) \\ & + 0.04d(n-5) + 0.03d(n-6) + 0.01d(n-7). \end{aligned} \tag{S4}$$

In the next step, this signal undergoes a nonlinear transformation subjected to Gaussian white noise with a signal-to-noise ratio of 32dB. The nonlinear transformation yield the reservoir input signal $u(n)$ with

$$u(n) = q(n) + 0.036q(n)^2 - 0.011q(n)^3 + v(n), \tag{S5}$$

where $v(n)$ denotes the Gaussian white noise. The task is to reconstruct the signal $d(n)$ from the reservoir input $u(n)$. The task is treated as a regression task as, e.g., in [5], to make the methods developed in this paper applicable. That is, we calculate the NMSE of predicting the target series $y(n)$ instead of symbol error rates.

2. CALCULATION OF THE RELATION BETWEEN IPCS AND TASK SPECIFIC NMSE

Consider a general fading memory task, to predict the values of a target function series $\hat{y}$ with

$$\hat{y}_m = \hat{f}(u_m^{-\infty}) + \xi_m, \tag{S6}$$

where $u_m^{-\infty} = (u_m, u_{m-1}, u_{m-2}, .., u_{m-\infty})$ is the series containing the "momentary" input u_m and all the inputs u_{m-i} prior to u_m, f is a fading memory function according to [6] and ξ_m is a noise sequence independent of the input. Later on, we neglect the noise part for simplification having $\hat{y}_m = f(u_m^{-\infty})$. Formally, we consider infinite input series here, but in practical situations the input series will be finite. As demanded f has fading memory, hence choosing suitable cut-off values is appropriate.
For convenience, define $\hat{\mathbf{y}} = (\hat{y}_1, \hat{y}_2, ...)^T, \hat{\mathbf{f}} := (\hat{f}(u_1^{-\infty}), \hat{f}(u_2^{-\infty}), ...)^T$ as the one-dimensional target vector and target function, respectively. It is often convenient to suppress the index m. That is $u^{-\infty} := (u_0, u_{-1}, u_{-2}, ...)$ denotes the sequence of all inputs prior to the "momentary" input u_0 and $\hat{y}(u^{-\infty})$ denotes the "momentary" target value both corresponding to index m.
Dambre et al. [6] showed, that any possible target function $f(u^{-\infty})$ on the Hilbert space of fading memory functions can be expressed in a corresponding basis $\hat{f}(u^{-\infty}) = \sum_n a_n P_n(u^{-\infty})$, where P_n denote the basis functions and n is an index, indexing the basis functions in an arbitrary order. In the main script and motivated by [6], we choose products of Legendre polynomials on i.i.d. uniform input in [-1,1] (because the complexity increases in a predictable manner which makes the evaluation of the IPCs practicable by choosing appropriate cutoffs, as described in Section 7), but other bases are possible as well. In vector notation, we find

$$\hat{\mathbf{y}} = \sum_n a_n \mathbf{P}_n + \xi, \tag{S7}$$

where $\mathbf{P}_n := (P_n(u_1^{-\infty}), P_n(u_2^{-\infty}), ...)^T$ is the vector containing basis function n evaluated at all entities of the input sequence.
In [7] it was shown, that the capacity $C_{\hat{y}}$ to reconstruct a target vector $\hat{\mathbf{y}}$ can be expressed as

$$C_{\hat{y}} = \frac{\hat{\mathbf{y}}^{\mathbf{T}}\mathbf{y}}{||\hat{\mathbf{y}}||^2}, \tag{S8}$$

where $\mathbf{y} = (y_1, y_2, ...)^T$ is the prediction of the target. Following Dambre et al. [6], the connection between the normalized root-mean-square error (NRMSE) and the capacity to compute $\hat{y}$ is

$$NMSE = \frac{(1 - C_{\hat{y}})\frac{||\hat{\mathbf{y}}||^2}{N_I}}{var(\hat{\mathbf{y}})}, \tag{S9}$$

where N_I is the number of inputs. Note, that we are interested in the testing prediction $\mathbf{y}_{test}$ and not into the prediction of the training data here.
In order to use Eq. (S9) to obtain an estimate for the NMSE, we need an expression for the capacity $C_{\hat{y}}$. Therefore, we evaluate Eq. (S8) using Eq. (S7) yielding

$$C_{\hat{y}} = \frac{\hat{\mathbf{y}}^{\mathbf{T}}\mathbf{y}}{||\hat{\mathbf{y}}||^2} = \frac{(\sum_n a_n \mathbf{P}_n)^T \mathbf{y}}{||\hat{\mathbf{y}}||^2} + h(\xi) = \sum_n a_n \frac{\mathbf{P}_n^T \mathbf{y}}{||\hat{\mathbf{y}}||^2} + h(\xi). \tag{S10}$$

Here, we denote with $h(\xi)$ the noise terms corresponding to the noise ξ_m in the target series, that can not be predicted out of the input. For the sake of simplicity, we will neglect all noise terms in the following calculations. The aim is to now develop the prediction $\mathbf{y}$ into the basis $\{P_n\}$ as

well. Therefore, we use the definition of the prediction $\mathbf{y} = \mathbf{S}\mathbf{w}$. Here, $\mathbf{S}$ is the (testing) state matrix to the given input and $\mathbf{w} = [(\mathbf{S}_{train}^T \mathbf{S}_{train})^{-1} \mathbf{S}_{train}^T]\hat{\mathbf{y}}_{train}$ is the solution for the weights vector of the linear regression, where $\mathbf{S}_{train}$ is the training state matrix. Note, that we choose un-regularised linear regression weights here (i.e. $\zeta = 0$ in the main body of the paper) in order to make the folowing analytic deviation possible. We can now develop the training target into Legendre polynomials $\hat{\mathbf{y}}_{train} = \sum_j a_j \mathbf{P}_j + \xi$ as above and obtain neglecting the noise

$$\mathbf{w} = [(\mathbf{S}_{train}^T \mathbf{S}_{train})^{-1} \mathbf{S}_{train}^T] \sum_j a_j \mathbf{P}_j = \sum_j a_j \mathbf{w}_j, \tag{S11}$$

where $\mathbf{w}_j = [(\mathbf{S}_{train}^T \mathbf{S}_{train})^{-1} \mathbf{S}_{train}^T]\mathbf{P}_j$ are the weights corresponding to the prediction of the jth basis function. This yields

$$\mathbf{y} = \sum_j a_j \mathbf{S}\mathbf{w}_j = \sum_j a_j \mathbf{p}_j,$$

where $\mathbf{p}_j$ is the prediction of the jth Legendre polynomial. We therefore developed the prediction of the target into predictions of the basis functions. Neglecting the noise terms $h(\xi), g(\xi)$, this yields with Eq. (S10)

$$\begin{aligned} C_{\hat{y}} &\approx \frac{\sum_n a_n \mathbf{P}_n^T \sum_j a_j \mathbf{p}_j}{||\hat{\mathbf{y}}||^2} = \sum_{n,j} a_n a_j \frac{\mathbf{P}_n^T \mathbf{p}_j}{||\hat{\mathbf{y}}||^2} = \sum_{n,j} a_n a_j \frac{< \mathbf{P}_n, \mathbf{p}_j >}{||\hat{\mathbf{y}}||^2} \\ &= \sum_n a_n^2 \frac{< \mathbf{P}_n, \mathbf{p}_n >}{||\hat{\mathbf{y}}||^2} \frac{||\mathbf{P}_n||^2}{||\mathbf{P}_n||^2} + \sum_n \sum_{j \neq n} a_n a_j \frac{< \mathbf{P}_n, \mathbf{p}_j >}{||\hat{\mathbf{y}}||^2} \frac{||\mathbf{P}_n||^2}{||\mathbf{P}_n||^2} \\ &= \sum_{n,j} a_n a_j C_{nj} \frac{||\mathbf{P}_n||^2}{||\hat{\mathbf{y}}||^2}, \end{aligned} \tag{S12}$$

where we introduced $C_{nj} := \frac{<\mathbf{P}_n, \mathbf{p}_j>}{||\mathbf{P}_n||^2}$. To connect this expression with the information processing capacity we assume, that the input series $\{u_0, u_1, ...\}$ is drawn from the same probability distribution as in the definition of the IPC, i.e., i.i.d. random numbers in [-1,1]. Under this assumption, the matrix $C_n j$ has the following interesting properties:
let IPC_n be the capacity to reconstruct the nth basis polynomial of the Hilbert space of fading memory functions according to Eq. (S7). Then the diagonal elements $C_{nn} = IPC_n$ are the information processing capacities to reconstruct the basis functions P_n and $Tr(C_{ij}) = \sum_n IPC_n$ is the total information processing capacity. The matrix C_{nj} can be interpreted as the IPC "confusion matrix", since the off diagonal elements are given by the scalar product between the prediction of one basis function and the other basis functions. However, one can easily see that for all n, j with $j \neq n$: $C_{nj} \leq IPC_n$ and if $IPC_n \lessapprox 1$ then $C_{nj} \approx 0$, for all $j \neq n$. This strongly limits the number of possibly relevant off-diagonal elements. Further, for i.i.d. input series, numerical analysis suggest, that the off diagonal elements vanish. Therefore, we neglect these terms in the following, that is we assume, the prediction p_n of polynomial P_n is statistically independent to the other orthogonal basis polynomials $P_j, j \neq n$.

Under this assumtions we obtain with Eq. (S12) and Eq. (S9) as an estimate for the NMSE:

$$\begin{aligned} NMSE &\approx \frac{||\hat{\mathbf{y}}||^2}{N_I \text{var}(\hat{\mathbf{y}})} - \frac{\sum_n a_n^2 ||\mathbf{P}_n||^2 IPC_n}{N_I \text{var}(\hat{\mathbf{y}})} \\ &= 1 - \sum_n \frac{a_n^2 ||\mathbf{P}_n||^2}{||\hat{\mathbf{y}}||^2} IPC_n. \end{aligned} \tag{S13}$$

In the last step we assumed that the mean of the target equals 0. A non-zero mean value can always be subtracted from the target values, however. Additionally, a constant bias term in the linear regression takes care of mean values unequal to one. The latter formula is therefore sufficient.

In the above derivation it was assumed that the input sequence is drawn from the same distribution as in the calculation of the IPC, i.e., i.i.d. values in [-1,1]. This is e.g., the case for NARMA task(s), if one shifts the input to the interval [-1,1]. It is not valid for arbitrary

tasks, however. In the main body of the paper, it is investigated, how Eq. (S13) performs if this assumption is violated. If the input series is not too high (auto-)correlated, one can still get a high correlation between Eq. (S13) and the simulated NMSE for input series from different distributions. See for example the Lorenz task results in the main script. Note, that Eq. (S13) is a weighted sum of IPCs:

$$NMSE \approx 1 - \sum_n c_n \cdot IPC_n. \tag{S14}$$

The coefficients $c_n = \frac{a_n^2 ||\mathbf{P}_n||^2}{||\hat{\mathbf{y}}||^2}$ therefore weight the importance of the individual information processing capacities IPC_n. However, Eq. (S14) is more general than the specific expression Eq. (S13). It could be used as a starting point for future measures choosing weights c_n better suited for non i.i.d. input distributions.
Note, that Eq. (S12) is developed for unregularised linear regression. However, introducing a regularisation like ridge regularisation is more natural from a practical point of view. In the main body of the paper, we therefore test Eq. (S12) against task specific NMSEs obtained with a small regularisation parameter ($\zeta = 10^{-6}$). Despite this deviation from the analytical assumptions Eq. (S12) works very well if the assumption of i.i.d. input is fulfilled (see main body of the paper). Setting the Tikonov regularisation Parameter to zero yields similiar results, nevertheless.

Remark on the norms of basis polynomials

For i.i.d. input and a Legendre polynomial basis, $P_n(u^{-\infty}) = \Pi_i p_{d_n^i}(u_{-i})$ as in the main body of the paper, one can evaluate the norms $||\mathbf{P}_n||$ of the basis functions analytically. Via evaluating the corresponding expressions and comparing them to a Monte-Carlo integration, one finds $\frac{||\mathbf{P}_n(u^{-\infty})||^2}{N_I} = \frac{1}{2^{N_n}} \Pi_i \int_{-1}^{1} p_{d_n^i}^2(u_{-i}) du_{-i} = \Pi_i^{N_n} \frac{1}{2d_i+1}$, where N_n is the number of individual Legendre polynomials involved in basis function P_n (d_i are the corresponding orders).

3. EVALUATION OF THE DEVELOPMENT COEFFICIENTS

It remains to determine the expansion coefficients a_n of Eq. (S7). For the evaluation of these coefficients to be put into Eq. (S13), we use a nonlinear vector regressive model of the form

$$\mathbf{y} = \sum_n \tilde{a}_n \mathbf{P}_n(u^{-\infty}). \tag{S15}$$

This is a multilinear regression model with state Matrix: $\mathbf{X} = [\mathbf{P}_1, \mathbf{P}_2, ...]$. The solution for the linear regression weights $\tilde{a}_n$ are an estimator for the searched coefficients a_n. To avoid over-fitting, a ridge regression with a regularization factor of 0.1 is used to solve the model in the main manuscript. Other types of regularization would be possible as well, nonetheless. Note that NVAR models become ambiguous if the input is correlated. It is known that multilinear models may yield bad estimates for the regression coefficients if the regressors, i.e., the $\mathbf{P_n}$ are strongly correlated. For e.g., the NARMA10 series input, the P_n are uncorrelated by construction, whereas for the Mackey-Glass task they are highly correlated (see main body of the paper). In all cases, a ridge regression coefficient of 0.1 was used. Note, that for practical evaluation of Eq. (S15) cut-off orders have to be chosen in order to evaluate a finite Number of coefficients. See Sec. 7 for a discussion.

4. SIMULATION DETAILS

Numerical simulations for the reservoir computing part were done in C++ using standard libraries and the linear algebra library "Armadillo" [8]. The delayed Stuart Landau equations (see main body of the manuscript) were integrated using a Runge-Kutta 4th order algorithm with $\Delta t = 0.01$. The matrix inverse was calculated via the Moore-Penrose-Pseudoinverse from the c++ linear algebra library "Armadillo". For all simulations, a time of 1000τ without input to let transients decay and a buffer time of 1000 inputs were simulated before the actual training was done.
In order to obtain the NMSE for the time series prediction tasks, 5000 input values were used in the training state to train the weights. For the IPC calculations, 40000 input values were taken.

In order to better connect to the measure of information processing capacity (IPC), before feeding the input into the reservoir, it is being normalised via a linear transformation, such that it lays approximately in the interval $[-1, 1]$. This ensures, that the input values come from the same

Table S1. Default parameter values for numerical simulations

Parameter	Description	value	Parameter	Description	value
τ	Delay	425	T	Clock cycle	200
λ	Pump rate	0.01	κ	Feedback strength	0.18
ϕ	Feedback phase	0	η	Input strength	0.06
α	Sheer parameter	0	ω	Frequency	0
γ	Nonlinearity	-0.1	N_v	virtual nodes per oscillator	100

range as in the definition of the IPC (see above) and also ensures that inputs do not become too large. For the NARMA10 input, which is i.i.d. distributed in [0,0.5] this is exactly possible. For the other input distributions, we simply subtract the mean value of the input sequence $\{u\}$ and divide through two standard deviations, i.e. $u_i \rightarrow \frac{u_i - \text{mean}(\{u\})}{2 \cdot \text{stddev}(\{u\})}$.

To avoid over-fitting, an additional regularization with noise was done for the time-series prediction tasks. Therefore, the Tickonov regularization coefficient was set to $\zeta = 10^{-6}$. The testing procedure involved 5000 inputs. For the masks, random binary values in $\{0, 1\}$ were chosen. The system parameters used for simulation are given in table S1 and correspond to our recent publication [9].

5. RELATION BETWEEN RESERVOIR COMPUTING AND NVAR MODELS

In this section, we show, that a reservoir computer can be viewed as an approximate nonlinear vector (auto)-regressive model. Consider an RC with a state matrix constructed out of N nodes $\mathbf{x} := (x^1, x^2, .., x^N)^T$. Since a reservoir computer fulfils the fading memory property, we can develop the node states x^j into a basis $P_n(u^{-\infty})$ on the Hilbert space of fading memory functions:

$$\mathbf{x} = \sum_{n=1}^{\infty} \mathbf{a}_n P_n(u^{-\infty}) + \boldsymbol{\xi}, \tag{S16}$$

where $\mathbf{a}_n$ denotes the weights vector corresponding to basis element P_n and $\boldsymbol{\xi}$ denotes a noise term independent of the input. The prediction y of a reservoir is a linear combination of the node states: $y =< \mathbf{w}, \mathbf{x} >$, where $\mathbf{w}$ denotes the weights vector. Combining that with Eq. (S16) yields

$$y = \left\langle \mathbf{w}, \sum_{n=1}^{\infty} \mathbf{a}_n P_n(u^{-\infty}) \right\rangle + \xi \tag{S17}$$

$$= \sum_{n=1}^{\infty} \langle \mathbf{w}, \mathbf{a}_n \rangle P_n(u^{-\infty}) + \xi = \sum_{n=1}^{\infty} \alpha_n(\mathbf{w}) P_n(u_m^{-\infty}) + \xi. \tag{S18}$$

Here $\alpha_n(\mathbf{w}) := \langle \mathbf{w}, \mathbf{a}_n \rangle$. The result is, that the reservoirs' prediction is a linear combination of the basis elements with coefficients $\alpha_n(\mathbf{w})$ plus a noise term. This is a nonlinear vector regressive model, where the coefficients α_n cannot be set independently. If the task is auto-regressive, it is a nonlinear vector regressive model (NVAR). As advantage, one has only to train N weights $w_1, w_2, ..., w_N$, instead of all α_n weights. If the infinite series in Eq. (S16) is truncated, one can obtain a system of linear equations to determine the weights $\mathbf{w}$ in dependence of the nvar factors α_n or vice versa. Note that for a linear reservoir, it has explicitly been shown, that reservoir computing is equivalent to vector auto-regressive models [10].

6. AUTO-CORRELATION FUNCTIONS

In The main body of the paper, we show the absolute auto-correlation function for the Lorenz x, Mackey-Glass and Channel Equalization input. The auto-correlation function is defined as:

$$\psi(j) = \sum_{i}^{N} \frac{\bar{y}_i \bar{y}_{i-j}}{N\text{var}(\bar{y})}, \tag{S19}$$

where $\bar{y}_i = y_i - \text{mean}(y)$ is the mean free target series. We evaluated the auto-correlation function with the python "statsmodels" library using N=40000 values.

7. CHOOSING APPROPRIATE CUT-OFF ORDERS

For concrete evaluation of Eq. (S13), one has to introduce suitable cut-off orders for polynomial order and previous input steps to be evaluated. Generally, one wants to evaluate as few terms as possible. The sufficient number of terms depend both on the task's series expansion Eq. (S7) and the number of IPCs a reservoir computer of interest is able to reconstruct. It is unnecessary to evaluate coefficients a_n, corresponding to IPC_ns that cannot be reconstructed by a RC. The memorizing capabilities of a delay based RC are mainly determined by the values of the delay time [9], one can therefore limit the number of previous steps accordingly. Apart from that, one should evaluate all terms with significant expansion coefficients in Eq. (S7).

Unfortunately, with increasing polynomial cut-off order, the number of terms increases exponentially ("curse of dimensionality"). That limits the possible cut-off orders. This is especially true for the nvar approach Eq. (S15) for calculating the coefficients a_n, since for a very high amount of regression vectors, overfitting issues occur.
The methods developed in this paper are therefore limited to tasks that can be well described with low order polynomial expansions, tasks where only the first few steps of previous inputs are necessary to reconstruct, e.g., nearly markovian time-serieses (see the Lorenz task example in the main body of the Paper) or tasks of which one has additional knowledge on the relevant terms, e.g., the NARMA10 task.

To obtain appropriate cut-off orders, we increase the cut-off orders incrementally. If the computational error of the above defined polynomial nvar model does not decrease any longer, we fix the cut-off orders to the given values. In the construction of Eq. (S15), one can choose cut-off values, such that only coefficients a_n corresponding to IPC_ns the RC setup of interest can reconstruct are evaluated, e.g., exploiting knowledge on the memorizing capabilities of a RC setup. However, it can be sufficient to consider a smaller number of a_n, if additional knowledge of the task is available.
For instance, in the nonlinear Channel Equalisation example, one knows per definition that only 10 previous input steps are relevant. As another example, the NARMA10 task definition explicitly shows the relevant previous input steps and nonlinear transformations on them and cut-offs can be chosen accordingly.

As a result, for Eq. (S13), we evaluated for the NARMA10 task capacities up to 2nd order and up to 20 previous inputs (including the "momentary" input), for the Lorenz task polynomials up to order 5 and corresponding up to 6 previous inputs and for the Channel Equalisation task, capacities up to third order and corresponding up to 10 steps into the past. For the Mackey Glass task, we evaluated up to 20 previous inputs and polynomials up to 3 order.

8. COMMENT ON NEGATIVE VALUES OF PREDICTED NMSE

In the main body of the Paper, it can be seen, that the predicted NMSE can exhibit negative values, if the assumptions of the derivation are not strictly fulfilled. Here we give a small explanation, why this can happen and doing so give an instructive example on how an accurate prediction for uncorrelated input series may break down for highly correlated input series.

Consider an ideal reservoir computer, that is perfect in terms of predictions of target values, i.e. all IPC_n equal 1 and the error the prediction is always 0. Then the Eq. (S13) becomes

$$NMSE \approx 1 - \frac{\sum_n a_n^2 ||\mathbf{P}_n||^2}{N_I \text{var}(\hat{\mathbf{y}})}. \tag{S20}$$

Now consider a simple target function that linearly depends on the last two previous inputs with zero mean value, i.e. $\hat{y} := ap_1(u_0) + ap_1(u_{-1})$, where $p_1(x) \equiv x$ denotes the first order Legendre polynomial. This target function has a variance of $\text{var}(\hat{y}) = a^2 var(u_0) + b^2 var(u_{-1}) + 2ab\text{Cov}(u_{-n}, u_{-j})$. For simplicity assume $\text{var}(u_{-1}) = \text{var}(u_0) =: v$ [1] and w.l.o.g. $\text{mean}(u_0) = \text{mean}(u_{-1}) = 0$. Both conditions are, e.g., fulfilled for i.i.d. distributed inputs. With this

[1] this should in fact always be the case for time autonomous input series

construction, we have $\frac{||\mathbf{u_0}||^2}{N_I} = \frac{||\mathbf{u_{-1}}||^2}{N_I} = v$. Putting this into the equation yields:

$$NMSE \approx 1 - \frac{(a^2+b^2)v}{\mathrm{var}(\hat{\mathbf{y}})} = 1 - \frac{(a^2+b^2)v}{(a^2+b^2)v + 2ab\mathrm{Cov}(u_0, u_{-1})} \tag{S21}$$

As one can see, this becomes 0 if the u_0, u_{-1} are independent, i.e. $\mathrm{Cov}(u_0, u_{-1}) = 0$. This means for independent input distributions, the predicted NMSE for this task becomes 0, exactly the true value for the perfect reservoir computer. However, if we choose a correlated input series, the covariance becomes finite and the NMSE prediction becomes imprecise, with increasing deviations for increasing covariance values.
For $\mathrm{Cov}(u_0, u_{-1}) \neq 0$ one can also obtain negative predictions of NMSE, e.g. for input series $u = \{1, -1, 1, -1, ...\}$ and $a = 1, b = 0.5$.

Additionally to the covariances appearing for correlated input series, there is also a possible offset of the predicted NMSE due to the neglected noise terms. Noise appears, e.g., in the Channel E. task.

However, in Eq. (S20), the variance of the target appears only as a scaling factor in the predicted NMSE, meaning that different values of $\mathrm{var}(\hat{y})$ only linearly transform the predicted NMSE. The same holds for a possible offset. When comparing different setups of (imperfect) reservoir computers, Eq. (S13) therefore remains its predictive power in these cases, despite not giving the exact value of a single setup. Note, that this example can easily be generalised to more complex target functions.

REFERENCES

1. A. F. Atiya and A. G. Parlos, "New results on recurrent network training: unifying the algorithms and accelerating convergence," IEEE Trans. Neural Netw. **11**, 697–709 (2000).
2. E. N. Lorenz, "Deterministic nonperiodic flow," J. Atmos. Sci. **20**, 130 (1963).
3. M. C. Mackey and L. Glass, "Oscillation and chaos in physiological control systems," Science **197**, 287 (1977).
4. A. Argyris, "Photonic neuromorphic technologies in optical communications," Nanophotonics (2022).
5. S. Ortín and L. Pesquera, "Reservoir computing with an ensemble of time-delay reservoirs," Cogn. Comput. **9**, 327–336 (2017).
6. J. Dambre, D. Verstraeten, B. Schrauwen, and S. Massar, "Information processing capacity of dynamical systems," Sci. Rep. **2**, 514 (2012).
7. F. Köster, S. Yanchuk, and K. Lüdge, "Insight into delay based reservoir computing via eigenvalue analysis," J. Phys. Photonics **3**, 024011 (2021). ArXiv:2009.07928.
8. C. Sanderson and R. Curtin, "Armadillo: a template-based C library for linear algebra," JOSS **1**, 26 (2016).
9. T. Hülser, F. Köster, L. C. Jaurigue, and K. Lüdge, "Role of delay-times in delay-based photonic reservoir computing," Opt. Mater. Express **12**, 1214–1231 (2022). ArXiv 2112.11830.
10. E. M. Bollt, "On explaining the surprising success of reservoir computing forecaster of chaos? the universal machine learning dynamical system with contrast to var and dmd," Chaos **31**, 013108 (2021).
